# Physical activity and health-related quality of life among Chinese adolescents: a cross-sectional statistical path model involving sleep disturbance and depressive symptoms

**DOI:** 10.3389/fped.2026.1954434

**Published:** 2026-09-03

**Authors:** Mengxuan Liu

**Affiliations:** School of Physical Education, Jiangxi Institute of Technology, Nanchang, China

**Keywords:** adolescents, China, depressive symptoms, health-related quality of life, physical activity, sleep disturbance, statistical association

## Abstract

**Objective:**

Physical activity (PA) is positively associated with health-related quality of life (HRQoL) in adolescents, while sleep disturbance and depressive symptoms are also closely related to both PA and HRQoL. However, relatively few studies have examined these factors simultaneously, particularly among Chinese adolescents. This study examined the association between PA and HRQoL and assessed the separate and serial indirect associations involving sleep disturbance and depressive symptoms.

**Methods:**

A cross-sectional survey was conducted among 2,202 secondary-school students from 12 schools in China. Participants completed validated self-report measures of PA, sleep disturbance, depressive symptoms, and HRQoL. Pearson correlations were used to examine bivariate associations, and a serial multiple-mediator model (PROCESS Model 6) was applied to estimate direct and indirect associations. Statistical uncertainty was evaluated using 5,000 bootstrap resamples and bias-corrected 95% confidence intervals (CIs).

**Results:**

Higher PA was associated with lower sleep disturbance and depressive symptoms and with better HRQoL. Sleep disturbance was positively associated with depressive symptoms, whereas both sleep disturbance and depressive symptoms were inversely associated with HRQoL. The standardized total association between PA and HRQoL was 0.324 [95% CI (0.288, 0.358)], of which 0.095 was statistically accounted for by the combined indirect associations. Significant specific indirect associations were observed through sleep disturbance alone [*β* = 0.032, 95% CI (0.019, 0.046)], depressive symptoms alone [*β* = 0.046, 95% CI (0.034, 0.058)], and the sequential pathway involving sleep disturbance followed by depressive symptoms [*β* = 0.017, 95% CI (0.013, 0.022)]. The direct PA–HRQoL association remained significant after both statistical mediators were included [*β* = 0.229, 95% CI (0.191, 0.265)].

**Conclusions:**

PA, sleep disturbance, depressive symptoms, and HRQoL were closely interconnected among Chinese adolescents. Sleep- and mood-related factors statistically accounted for part of the positive association between PA and HRQoL, with depressive symptoms showing the largest specific indirect association. These findings support considering PA, sleep health, and emotional well-being within an integrated adolescent health framework. Because all variables were measured concurrently, the observed indirect associations should not be interpreted as evidence of temporal or causal mediation.

## 1 Introduction

### 1.1 Adolescent HRQoL and physical activity

Health-related quality of life (HRQoL) captures how individuals evaluate their physical, psychological, and social functioning in daily life. Unlike indicators focused on symptoms or disease status, HRQoL reflects a broader appraisal of well-being and functional capacity, making it particularly relevant to adolescent health research (1–5). Adolescence involves rapid biological maturation alongside substantial changes in emotional regulation, social relationships, and academic expectations. These transitions can alter how young people perceive their health and manage everyday demands. Within a biopsychosocial perspective, adolescent HRQoL is shaped by the interaction of health behaviors, sleep patterns, emotional functioning, and the surrounding school and family environments (6–10). Global evidence indicates that mental health problems affect a considerable proportion of adolescents, while sleep loss, academic strain, and insufficient physical activity (PA) frequently occur within the same population (11–13). These concerns may be especially salient in China, where examination pressure, competitive academic evaluation, and high-stakes educational transitions can reduce opportunities for recreation and recovery (14–17). Identifying modifiable factors associated with HRQoL is therefore important for understanding how adolescents maintain functioning under sustained developmental and academic demands (18–21).

**Figure 1:**
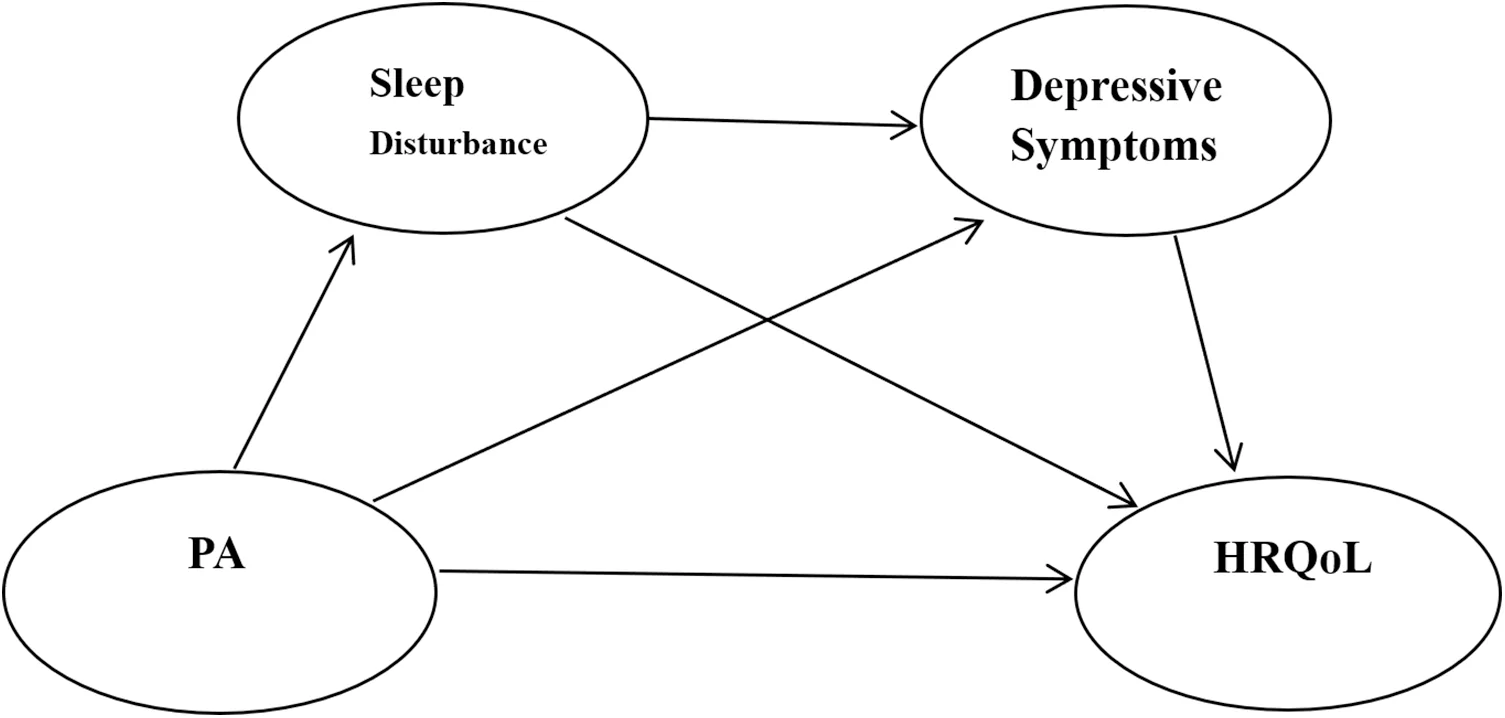
Hypothesized cross-sectional statistical path model of the associations among PA, sleep disturbance, depressive symptoms, and HRQoL. PA, physical activity; HRQoL, health-related quality of life. Arrows represent statistical regression paths specified within the cross-sectional model and do not imply temporal ordering or causal effects.

PA is one of the most accessible behavioral factors relevant to adolescent health (22–24). Current guidelines recommend an average of at least 60 min of moderate-to-vigorous physical activity per day for children and adolescents (25–28), yet adherence remains low in many settings (29–31). Higher PA has been associated with better physical fitness, greater vitality, stronger social participation (32–35), and more favorable psychological functioning (36–38). These benefits are closely aligned with the physical, emotional, and interpersonal dimensions assessed by HRQoL measures. PA may also provide opportunities for stress relief, perceived competence, and peer interaction, all of which can contribute to more positive evaluations of daily functioning. At the same time, low PA often co-occurs with sleep disturbance and depressive symptoms (39, 40). This pattern suggests that the relationship between PA and HRQoL may involve more than a direct association and may partly reflect the contribution of sleep-related and emotional factors (41, 42) (Figure 1).

### 1.2 Sleep disturbance and HRQoL

Sleep disturbance is common during adolescence and can affect attention, academic performance, emotional stability (43, 44), and perceived health (45). Adolescent sleep is influenced by both biological and environmental conditions. The two-process model of sleep regulation emphasizes the combined roles of homeostatic sleep pressure and circadian timing, whereas cognitive-behavioral accounts highlight the disruptive effects of physiological arousal, rumination, and stress-related cognition (46, 47). These processes may become more pronounced when school schedules, academic workload, and screen use interfere with regular sleep routines (48, 49). PA has been associated with better subjective sleep quality and lower levels of sleep disturbance in adolescent populations (50). Several explanations have been proposed, including greater daytime energy expenditure, more stable daily rhythms, and reduced stress-related arousal. Although the direction of this relationship cannot be determined from cross-sectional evidence, sleep disturbance remains a plausible statistical correlate through which PA and HRQoL may be connected.

### 1.3 Depressive symptoms and HRQoL

Depressive symptoms provide a second potential explanation for variation in adolescent HRQoL. Their consequences extend beyond low mood and may include reduced energy, poorer concentration, loss of interest, diminished academic participation, and withdrawal from social activities (51–53). Because HRQoL incorporates perceived functioning across several life domains, even subclinical depressive symptoms may be associated with lower overall scores (54, 55). Previous research has consistently identified negative relationships between depressive symptoms (56) and HRQoL among adolescents and other young populations (57, 58). PA is also commonly associated with fewer depressive symptoms, although the strength and direction of this relationship may vary according to study design and population characteristics (59, 60). More active adolescents may experience greater opportunities for social engagement, stress regulation, and mastery, whereas depressive symptoms may limit motivation and participation in activity. The present study does not attempt to establish which process occurs first, but the observed literature supports examining depressive symptoms as a statistical correlate within the PA–HRQoL relationship.

### 1.4 Serial indirect associations

Sleep disturbance and depressive symptoms are closely related rather than independent concerns. Sleep disturbance is associated with greater emotional reactivity, weaker cognitive control, and difficulty regulating negative affect, all of which may accompany depressive symptoms (61). Longitudinal studies have often found that sleep disturbance precedes later emotional problems, although reciprocal associations have also been reported. This evidence provides a theoretical basis for placing sleep disturbance before depressive symptoms in the proposed model, while not establishing that this ordering applies causally to the present data. Under this specification, PA is expected to be associated with lower sleep disturbance, lower sleep disturbance with fewer depressive symptoms, and fewer depressive symptoms with better HRQoL (62–64). The model therefore represents a theoretically informed arrangement of concurrent associations rather than a confirmed temporal sequence.

Much of the existing literature has examined PA, sleep, depressive symptoms, and HRQoL in separate analyses. Studies that consider these variables together remain comparatively limited, particularly among Chinese adolescents exposed to demanding school environments. Examining sleep disturbance and depressive symptoms within the same model may help determine whether they account for distinct portions of the PA–HRQoL association and whether an additional serial indirect association is present. This question has practical relevance because school-based health programs often address PA, sleep, and emotional well-being as separate targets despite their frequent co-occurrence. Evidence of interconnected associations would support a more integrated approach, although intervention effectiveness would still need to be established through longitudinal or experimental research.

### 1.5 The present study

The present study therefore examined the association between PA and HRQoL among Chinese adolescents and assessed the statistical roles of sleep disturbance and depressive symptoms. A serial mediation model was specified to estimate independent indirect associations through each variable and a serial indirect association involving both variables. Because the study used cross-sectional data, the proposed ordering was theory-based and should not be interpreted as evidence of temporal precedence or causality. The following hypotheses were examined:

**H1.** PA is positively associated with HRQoL among Chinese adolescents.
**H2.** PA is negatively associated with sleep disturbance among Chinese adolescents.
**H3.** Sleep disturbance is positively associated with depressive symptoms among Chinese adolescents.
**H4.** Depressive symptoms are negatively associated with HRQoL among Chinese adolescents.
**H5.** Sleep disturbance and depressive symptoms are expected to form a significant statistical association pattern within the specified model linking PA and HRQoL. Specifically, higher PA is associated with lower sleep disturbance, lower sleep disturbance is associated with fewer depressive symptoms, and fewer depressive symptoms are associated with better HRQoL. These associations represent relationships estimated within the statistical model rather than temporal processes.

## 2 Materials and methods

### 2.1 Participants

The survey was administered from April to June 2025 in 12 secondary schools located in a central province of China. Schools were included according to their accessibility and willingness to support the study. Eligible participants were students enrolled in Grades 7, 8, 10, or 11. Students in Grades 9 and 12 were not recruited because data collection coincided with their preparation for secondary-school or university entrance examinations.

To participate, students were required to be enrolled in one of the selected grades, fall within the expected age range for that grade, understand the questionnaire instructions, and complete the survey independently. Participation also required assent from the student and informed consent from a legal guardian. Approval was obtained from each participating school before recruitment commenced.

The study involved human participants. The study protocol was reviewed and approved by the Institutional Review Board of Jiangxi Institute of Technology (IRB-JXUT-PEC-20241208). Informed consent was obtained from the participants' legal guardians, and assent was obtained from all adolescent participants before data collection. All procedures were conducted in accordance with the Declaration of Helsinki. Participation was voluntary and anonymous, and students were informed that they could discontinue the survey at any point without penalty.

No formal *a priori* power calculation was used to determine the recruitment target. Recruitment was based on the number of eligible students accessible in the selected schools during the study period. A total of 2,400 questionnaires were distributed across the participating schools. The final analytic sample comprised 2,202 adolescents. A sensitivity calculation based on this final sample indicated that, with a two-sided *α* of.05% and 80% statistical power, the study could detect a bivariate correlation of approximately |r| = 0.06 or larger. The final sample was therefore sufficient to detect small bivariate associations, although statistical significance in this large sample should not be equated with clinical or practical importance.

A total of 2,298 completed questionnaires were returned, equivalent to 95.75% of the questionnaires distributed. Data screening was conducted before the statistical analyses. Thirty-two records were excluded because one or both attention-check items were answered incorrectly. An additional 21 questionnaires were removed because respondents selected the same response category across the entire survey, and 43 were excluded because substantial portions of the questionnaire were incomplete. Potentially unusual observations exceeding three standard deviations from the mean of the principal variables were examined individually. These observations had already been identified through the preceding quality-control criteria, and no additional cases were removed. The final analytic sample comprised 2,202 adolescents, yielding an effective response rate of 91.75%.

### 2.2 Measures

#### 2.2.1 Physical activity

PA was measured with the Chinese Physical Activity Rating Scale-3 (PARS-3), developed by Liang (65). The PARS-3 has been widely used in Chinese populations to assess habitual physical activity and exercise participation (66–68). The scale assesses exercise behavior according to intensity, duration, and frequency, with each dimension rated using five response categories. The composite score was computed using the following scoring rule: intensity × (duration − 1) × frequency. Possible scores range from 0 to 100, with larger values reflecting greater engagement in PA. According to the established classification criteria, scores from 0 to 19 indicate a low level of PA, scores from 20 to 42 indicate a moderate level, and scores from 43 to 100 indicate a high level. Cronbach's *α* was 0.73 in the present sample.

#### 2.2.2 Sleep disturbance

Subjective sleep disturbance during the preceding month was evaluated using the Pittsburgh Sleep Quality Index (PSQI) (69–71). The PSQI comprises seven components: subjective sleep quality, sleep latency, sleep duration, habitual sleep efficiency, sleep disturbances, use of sleep medication, and daytime dysfunction. Each component is scored from 0 to 3, and the seven component scores are summed to yield a global score ranging from 0 to 21. Higher scores indicate greater sleep disturbance. Cronbach's *α* was 0.88 in the present sample.

#### 2.2.3 Depressive symptoms

Depressive symptoms over the previous two weeks were assessed with the Patient Health Questionnaire-9 (PHQ-9) (72–74). Each of the nine items is scored from 0, corresponding to “not at all,” to 3, corresponding to “nearly every day.” For the present analyses, responses across the nine items were averaged, resulting in a score ranging from 0 to 3. Higher mean scores indicate more frequent or severe depressive symptoms. Internal consistency was good, with a Cronbach's *α* of 0.87.

#### 2.2.4 Health-related quality of life

HRQoL was assessed using the KIDSCREEN-10 questionnaire (75–77). The instrument consists of 10 items assessing adolescents' perceptions of physical and psychological well-being, autonomy, relationships with parents and peers, and functioning in the school context. Items are rated on a five-point response scale. After reverse-coding the relevant items so that higher values consistently indicated better HRQoL, the 10 item scores were averaged to obtain an overall score ranging from 1 to 5. Higher scores indicated more favorable HRQoL. The present study therefore used an item-mean score and did not apply the conventional Rasch-based T-score transformation. Cronbach's *α* was 0.80 in the present sample.

### 2.3 Data collection and quality assurance

Before formal data collection, all research assistants completed standardized training. The training covered the study protocol, communication with adolescent participants, questionnaire administration, informed-consent procedures, privacy protection, and secure data management.

The questionnaire was pilot-tested with 40 students who were not included in the final sample. The pilot assessment focused on whether the instructions, response options, and item wording were understandable to students within the target age range. Feedback obtained during this stage was used to improve the clarity of the survey administration procedures.

The formal survey was administered electronically during scheduled school sessions. Research assistants introduced the study using standardized instructions and remained present to answer procedural questions without directing or influencing participants' responses. Students were permitted to access the survey only after the required consent and assent procedures had been completed.

No directly identifying information was collected. Electronic survey records were encrypted and accessible only to authorized members of the research team. Two instructed-response items were positioned at different points in the questionnaire to detect inattentive responding. Before the dataset was finalized, records were also evaluated for missing responses and invariant answering patterns using criteria established prior to data analysis.

### 2.4 Statistical analysis

The theoretically specified indirect association model was evaluated using Model 6 of Hayes' PROCESS macro. PA was entered as the independent variable, sleep disturbance as the first statistical mediator, depressive symptoms as the second statistical mediator, and HRQoL as the dependent variable. The regression equations were estimated using ordinary least squares.

Standardized regression coefficients were reported with their corresponding standard errors, *t* values, *p* values, and 95% confidence intervals. Standardized estimates were also used for the total, direct, total indirect, and specific indirect associations, allowing the relative magnitudes of the pathways to be compared.

The statistical uncertainty of the indirect estimates was examined using 5,000 bootstrap resamples and bias-corrected 95% confidence intervals. An indirect association was considered statistically significant when the corresponding bootstrap confidence interval did not contain zero. The reported bootstrap standard errors and confidence intervals refer to the standardized indirect effect estimates.

Because all variables were assessed concurrently, the specified ordering of sleep disturbance and depressive symptoms was based on theory rather than observed temporal precedence. The model was therefore interpreted as a representation of cross-sectional statistical associations and not as evidence of causal mediation. All tests were two-tailed, and statistical significance was defined as *p* < .05.

## 3 Results

### 3.1 Common method bias test

An unrotated exploratory factor analysis including all measurement items was conducted as an initial assessment of common method variance. Five factors had eigenvalues exceeding 1. The first factor explained 35.72% of the total variance, which was below the conventional threshold, indicating that a single factor dominated the covariance among the measures. Nevertheless, this result should be regarded as preliminary, because Harman's single-factor procedure cannot fully eliminate the possibility of shared method variance.

### 3.2 Sample characteristics

The final sample comprised 2,202 adolescents recruited from 12 secondary schools in a central Chinese province. Participants had a mean age of 14.45 years (SD = 1.64). Grade 7 students constituted the largest subgroup, accounting for 39.6% of the sample, followed by Grade 10 students at 27.7% and Grade 8 students at 27.0%. Grade 11 students represented 5.7% of the participants. Regarding family residence, 56.5% reported living in rural areas and 43.5% in urban areas. Detailed characteristics are summarized in Table 1.

**Table 1:** Demographic characteristics of the study participants (*N* = 2,202).

Characteristics	Categories	*n* (%)	M ± SD
Grade	Grade 7	871 (39.6)	
	Grade 8	595 (27.0)	
	Grade 10	611 (27.7)	
	Grade 11	125 (5.7)	
Family residence	Rural	1,245 (56.5)	
	Urban	957 (43.5)	
Age			14.45 ± 1.64

### 3.3 Correlation analysis results

Table 2 presents the means, standard deviations, and Pearson correlation coefficients for the principal study variables. All observed PSQI global scores were within the permissible range of 0–21. Although the standard deviation was relatively large (SD = 8.14), verification of the seven component scores, global-score calculation, and descriptive statistics identified no coding, calculation, or data-entry errors. The relatively wide dispersion therefore reflects substantial between-participant variability in self-reported sleep disturbance within the study sample.

**Table 2:** Correlation analysis results.

Variables	M	SD	1	2	3	4
1. PA	24.91	27.92	1			
2. Sleep disturbance	10.77	8.14	−0.23***	1		
3. HRQoL	3.09	0.85	0.33***	−0.27***	1	
4. Depressive symptoms	0.61	0.56	−0.32***	0.43***	−0.33***	1

PA, physical activity; HRQoL, health-related quality of life*.*

*** *p* < .001.

PA showed inverse correlations with sleep disturbance (*r* = −0.23, *p* < .001) and depressive symptoms (*r* = −0.32, *p* < .001), whereas its correlation with HRQoL was positive (*r* = 0.33, *p* < .001). Sleep disturbance was positively correlated with depressive symptoms (*r* = 0.43, *p* < .001) and inversely correlated with HRQoL (*r* = −0.27, *p* < .001). Depressive symptoms were likewise negatively correlated with HRQoL (*r* = −0.33, *p* < .001). Across all variable pairs, the absolute correlation coefficients ranged from 0.23 to 0.43, indicating associations of small to moderate magnitude.

### 3.4 Direct association between physical activity and health-related quality of life

**Figure 2:**
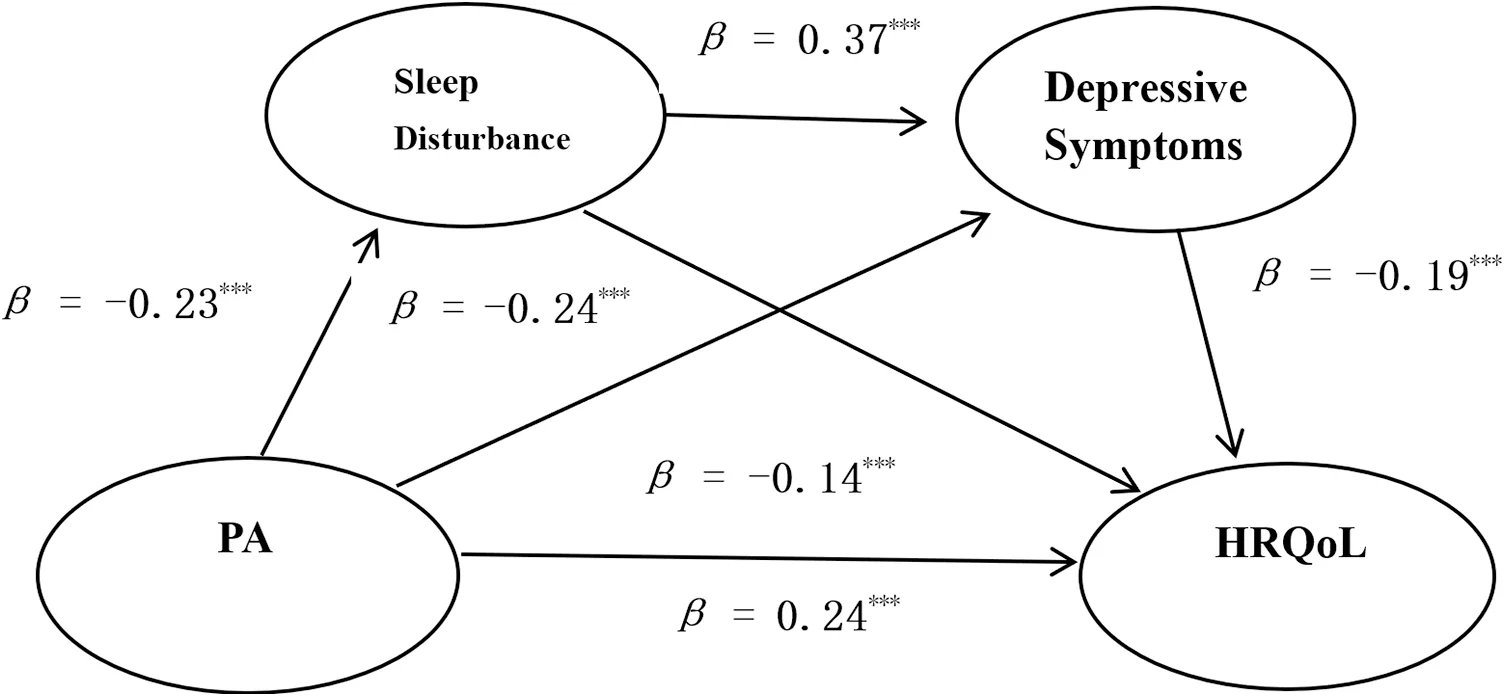
Standardized path coefficients for the hypothesized cross-sectional statistical path model. PA, physical activity; HRQoL, health-related quality of life. ***p < .001. The arrows represent statistical regression paths specified within the cross-sectional model and do not imply temporal ordering or causal effects.

The standardized regression estimates for the hypothesized serial indirect association model are reported in Table 3 and illustrated in Figure 2. Higher PA was related to lower sleep disturbance, with a standardized coefficient of *β* = −0.23 (*p* < .001). In the equation predicting depressive symptoms, both PA and sleep disturbance contributed significantly. PA was inversely associated with depressive symptoms (*β* = −0.24, *p* < .001), whereas sleep disturbance was positively associated with depressive symptoms (*β* = 0.37, *p* < .001).

**Table 3:** Regression analysis of the hypothesized serial indirect association model.

Dependent variable	Independent variable	*β*	SE	*t*	R^2^	*F*
Sleep disturbance	PA	−0.23	0.02	−10.86	0.05	117.87
Depressive symptoms	PA	−0.24	0.02	−12.40	0.23	335.69
	Sleep disturbance	0.37	0.02	19.37		
Health-related quality of life	PA	0.24	0.02	11.47	0.18	159.95
	Sleep disturbance	−0.14	0.02	−6.45		
	Depressive symptoms	−0.19	0.02	−8.79		

*β*, standardized regression coefficient; SE, standard error; PA, physical activity; HRQoL, health-related quality of life.

In the final equation predicting HRQoL, PA retained a positive association with HRQoL after sleep disturbance and depressive symptoms were entered into the model (*β* = 0.24, *p* < .001). By contrast, sleep disturbance (*β* = −0.14, *p* < .001) and depressive symptoms (*β* = −0.19, *p* < .001) were each inversely associated with HRQoL.

The regression equations accounted for 5% of the variance in sleep disturbance, 23% of the variance in depressive symptoms, and 18% of the variance in HRQoL.

### 3.5 Indirect associations through sleep disturbance and depressive symptoms

The total, direct, and specific indirect associations are presented in Table 4. The standardized total association between PA and HRQoL was 0.324, with a bootstrap SE of 0.018 and a 95% bootstrap CI of [0.288, 0.358]. After sleep disturbance and depressive symptoms were entered into the model, the standardized direct association remained statistically significant at 0.229, with a bootstrap SE of 0.019 and a 95% bootstrap CI of [0.191, 0.265]. The standardized total indirect association was estimated at 0.095, with a bootstrap SE of 0.008 and a 95% bootstrap CI of [0.079, 0.112].

**Table 4:** Total, direct, and specific indirect associations.

Path	Standardized effect	Boot SE	Boot LLCI	Boot ULCI	Effect proportion
Total	0.324	0.018	0.288	0.358	100.00
Direct	0.229	0.019	0.191	0.265	70.75
Total Indirect	0.095	0.008	0.079	0.112	29.25
Ind 1	0.032	0.007	0.019	0.046	9.83
Ind 2	0.046	0.006	0.034	0.058	14.17
Ind 3	0.017	0.002	0.013	0.022	5.24

Effects are reported as standardized effect estimates. Boot SE, bootstrap standard error; LLCI, lower limit of the bias-corrected 95% bootstrap confidence interval; ULCI, upper limit of the bias-corrected 95% bootstrap confidence interval; PA, physical activity; HRQoL, health-related quality of life. Indirect associations were evaluated using 5,000 bootstrap samples. Specific indirect estimates may not sum exactly to the total indirect estimate because of rounding.

Each of the three standardized specific indirect associations was statistically different from zero. The indirect association involving sleep disturbance alone was 0.032, with a bootstrap SE of 0.007 and a 95% bootstrap CI of [0.019, 0.046]. The corresponding estimate for depressive symptoms alone was 0.046, with a bootstrap SE of 0.006 and a 95% bootstrap CI of [0.034, 0.058]. The serial indirect association involving sleep disturbance followed by depressive symptoms was 0.017, with a bootstrap SE of 0.002 and a 95% bootstrap CI of [0.013, 0.022].

Among the specific indirect associations, the pathway involving depressive symptoms alone produced the largest standardized estimate. Because the direct PA–HRQoL association remained statistically significant after both statistical mediators were included, sleep disturbance and depressive symptoms statistically accounted for part, but not all, of the observed association. Given the concurrent measurement of all variables, these estimates should be understood as model-based indirect associations rather than evidence that PA temporally influenced sleep disturbance, depressive symptoms, or HRQoL.

## 4 Discussion

This study examined the relationships among PA, sleep disturbance, depressive symptoms, and HRQoL in Chinese adolescents. Adolescents who reported greater PA generally had better HRQoL and lower levels of sleep disturbance and depressive symptoms. Sleep disturbance was related to more depressive symptoms, and higher scores on both variables corresponded to poorer HRQoL. The bootstrap analysis also identified indirect associations involving sleep disturbance and depressive symptoms, both separately and in the order proposed by the theoretical model. Because the data were collected at one time point, the results describe concurrent relationships and do not establish how these variables develop over time.

Greater PA was associated with more favorable HRQoL, which accords with earlier evidence that active adolescents tend to report better physical, psychological, and social functioning (78, 79). HRQoL reflects not only the absence of illness but also adolescents’ perceptions of energy, emotional well-being, interpersonal relationships, and everyday functioning. Participation in PA may be relevant to these experiences because it is often accompanied by greater physical vitality, stronger perceptions of competence, more frequent peer interaction, and opportunities to relieve academic or emotional stress (80–83).

The association between PA and HRQoL remained significant after sleep disturbance and depressive symptoms were considered. This result indicates that these two variables captured only part of the observed relationship. Other characteristics not included in the analysis may also be relevant. For example, physically active adolescents may have better fitness, stronger self-esteem, wider social networks, or more structured daily routines (84, 85). Each of these characteristics could influence how adolescents evaluate their health and functioning.

PA was inversely associated with sleep disturbance. Similar patterns have been reported in studies of adolescent PA and subjective sleep outcomes (86, 87). One possible interpretation is that greater daytime activity strengthens sleep pressure and promotes more regular daily rhythms. PA may also coincide with less sedentary time, reduced evening screen exposure, or lower stress-related arousal. However, the relationship may also operate in the opposite direction. Adolescents experiencing greater sleep disturbance may have less energy and motivation to participate in PA (88, 89). Academic workload, family routines, and screen use may additionally influence both sleep and PA. The current data cannot distinguish among these possibilities.

Sleep disturbance was positively associated with depressive symptoms. This result is compatible with evidence that sleep disturbance and emotional distress often occur together during adolescence (90). Sleep disturbance may be accompanied by stronger emotional reactivity, weaker cognitive control, and greater difficulty managing negative affect. At the same time, depressive symptoms can interfere with sleep onset, continuity, and daily routines (91). Sleep disturbance was placed before depressive symptoms in the model because this ordering was supported by the theoretical framework and some longitudinal evidence. Nevertheless, the analysis does not show that sleep disturbance preceded depressive symptoms in this sample.

Depressive symptoms were related to poorer HRQoL in both the correlation and regression analyses. Symptoms such as fatigue, reduced interest, concentration difficulties, and withdrawal from school or social activities may affect several aspects of everyday functioning. Their relationship with HRQoL is therefore unlikely to be restricted to emotional well-being. Depressive symptoms may also accompany less favorable perceptions of physical health, family relationships, peer participation, and school experiences (92, 93). Conversely, persistent difficulties in these areas may contribute to emotional distress (94). The direction of this relationship cannot be determined from the current design.

The bootstrap estimates showed that sleep disturbance and depressive symptoms each contributed to the indirect association between PA and HRQoL. An additional indirect association was observed when sleep disturbance and depressive symptoms were considered in sequence (95). In the proposed model, greater PA corresponded to fewer sleep disturbances, fewer sleep disturbances corresponded to fewer depressive symptoms, and fewer depressive symptoms corresponded to better HRQoL. This pattern indicates that behavioral, sleep-related, and emotional factors were interconnected in the study sample. It does not demonstrate that increasing PA would subsequently improve sleep, reduce depressive symptoms, or enhance HRQoL. Longitudinal studies are needed to compare the proposed ordering with reverse and reciprocal relationships.

Related research conducted among Chinese university students and emerging adults has also indicated that PA, sleep, emotional functioning, family context, and digital behavior may be interconnected. PA has been associated with lower levels of problematic mobile phone use and negative emotion, whereas stress, smartphone addiction, loneliness, psychological resilience, and depressive symptoms have been examined as potential explanatory or modifying factors in associations involving PA and sleep-related outcomes (96–101). These studies broaden the range of plausible behavioral and psychological pathways underlying the present findings. However, because they predominantly involved university students or emerging adults and examined outcomes that differed from HRQoL, their findings should be generalized to secondary-school students with caution.

The direct association between PA and HRQoL remained significant after the two statistical mediators were entered. This suggests that a broader range of physical, psychological, and social characteristics may be involved. Future research could examine selected variables such as physical fitness, self-efficacy, social support, sedentary behavior, screen exposure, and academic stress. These extensions should be guided by a clear theoretical rationale rather than by adding a large number of overlapping variables to the same model.

The results may also be relevant to adolescent health promotion, although they do not demonstrate that a particular intervention would be effective. PA, sleep health, and emotional well-being are often addressed separately in school settings. The observed relationships indicate that they may be more appropriately considered as connected aspects of adolescent health. Schools could combine accessible opportunities for enjoyable PA with education about regular sleep routines and procedures for identifying persistent sleep or emotional difficulties. The value of such an integrated approach should be tested in prospective and intervention studies.

### 4.1 Strengths and limitations

This study has several strengths. It included a large sample of 2,202 adolescents recruited from 12 secondary schools and examined PA, sleep disturbance, depressive symptoms, and HRQoL within a single statistical framework. The study used established instruments, incorporated prespecified data-quality checks, and reported standardized estimates with bootstrap confidence intervals. It also distinguished the separate and sequential indirect associations involving sleep disturbance and depressive symptoms. In addition, the interpretation was deliberately aligned with the cross-sectional design, avoiding claims of temporal or causal mediation.

Several limitations should be considered when interpreting the findings. First, all variables were assessed at a single time point. The cross-sectional design therefore cannot establish whether physical activity preceded sleep disturbance, whether sleep disturbance preceded depressive symptoms, or whether the observed associations were directional or causal. Although the ordering of the two statistical mediators was theoretically informed, reverse, reciprocal, and alternative model specifications remain plausible. Accordingly, the indirect estimates should be interpreted as associations generated by the specified statistical model rather than as evidence of temporal or causal mediation.

Second, all measures were based on participants' self-reports. Responses may have been affected by recall bias, socially desirable responding, and shared method variance. Although Harman's single-factor analysis did not identify a single dominant factor, this finding does not eliminate the possibility of common method bias. In addition, the PARS-3 provides only a broad estimate of physical activity and does not distinguish among activity settings or specific types of exercise, nor does it provide an objective assessment of activity intensity. Future studies could combine self-report questionnaires with accelerometry, objective sleep measures, clinical interviews, and reports from parents or teachers.

Third, participants were recruited through convenience sampling from 12 secondary schools in one central Chinese province. The findings may therefore not be generalizable to adolescents from other geographical regions, socioeconomic backgrounds, educational systems, or cultural contexts. Although age, grade, and family residence were available and descriptively reported, these variables were not included as covariates in the primary statistical models. Residual confounding associated with these demographic characteristics therefore cannot be excluded. Moreover, the analyses did not explicitly account for the clustering of students within the 12 participating schools. Potential within-school dependence may consequently have affected the precision of the estimated standard errors. Future studies should recruit more geographically and socioeconomically diverse samples and use multilevel models or cluster-robust estimation to account for the hierarchical structure of students nested within schools.

Fourth, several potentially relevant characteristics were not measured or comprehensively assessed, including sex, body mass index, socioeconomic status, academic stress, screen use, chronic health conditions, family characteristics, and school environmental factors. The absence of these variables limited our ability to evaluate their potential confounding or moderating roles. Future research should incorporate a broader range of individual-, family-, and school-level characteristics to provide a more comprehensive assessment of the associations among physical activity, sleep disturbance, depressive symptoms, and HRQoL.

Finally, although the large sample provided relatively precise estimates, several observed associations were small or moderate in magnitude. Statistical significance should therefore not be interpreted as evidence of substantial clinical or practical importance. Longitudinal studies incorporating repeated assessments, objective measures, broader covariate adjustment, and appropriate analysis of the clustered sampling structure are needed to examine these associations more rigorously.

## 5 Conclusions

Among the Chinese adolescents included in this study, greater PA was associated with better HRQoL and lower levels of sleep disturbance and depressive symptoms. Sleep disturbance and depressive symptoms contributed to separate and sequential indirect associations between PA and HRQoL, while the direct association remained significant. These results indicate that PA, sleep health, emotional well-being, and HRQoL were closely related within the study sample. However, the cross-sectional design does not establish temporal order or causal mediation. Longitudinal and experimental evidence is needed before the proposed sequence can be interpreted as a developmental process or a basis for intervention.

## Data Availability

The raw data supporting the conclusions of this article will be made available by the authors, without undue reservation.
